# Identification and characterization of immature *Anopheles* and culicines (Diptera: Culicidae) at three sites of varying malaria transmission intensities in Uganda

**DOI:** 10.1186/s12936-020-03304-7

**Published:** 2020-06-23

**Authors:** Alex K. Musiime, David L. Smith, Maxwell Kilama, Otto Geoffrey, Patrick Kyagamba, John Rek, Melissa D. Conrad, Joaniter I. Nankabirwa, Emmanuel Arinaitwe, Anne M. Akol, Moses R. Kamya, Grant Dorsey, Sarah G. Staedke, Chris Drakeley, Steve W. Lindsay

**Affiliations:** 1 Infectious Diseases Research Collaboration, Kampala, Uganda; 2 Department of Zoology, Entomology and Fisheries Sciences, College of Natural Sciences, Makerere University, Kampala, Uganda; 3 Department of Health Metrics Sciences, University of Washington, Seattle, USA; 4 Department of Medicine, Makerere University College of Health Sciences, Kampala, Uganda; 5 Department of Medicine, San Francisco General Hospital, University of California, San Francisco, USA; 6 Faculty of Infectious and Tropical Diseases, London School of Hygiene and Tropical Medicine, London, UK; 7 Department of Immunology and Infection, London School of Hygiene and Tropical Medicine, London, UK; 8 Department of Biosciences, Durham University, Durham, UK

**Keywords:** *Anopheles*, Culicine, Anopheline, Larvae, Pupae, Aquatic habitats, Malaria, Uganda

## Abstract

**Background:**

Over the last two decades, there has been remarkable progress in malaria control in sub-Saharan Africa, due mainly to the massive deployment of long-lasting insecticidal nets and indoor residual spraying. Despite these gains, it is clear that in many situations, additional interventions are needed to further reduce malaria transmission. The World Health Organization (WHO) has promoted the Integrated Vector Management (IVM) approach through its Global Vector Control Response 2017–2030. However, prior roll-out of larval source management (LSM) as part of IVM, knowledge on ecology of larval aquatic habitats is required.

**Methods:**

Aquatic habitats colonized by immature *Anopheles* and culicines vectors were characterized at three sites of low, medium and high malaria transmission in Uganda from October 2011 to June 2015. Larval surveys were conducted along transects in each site and aquatic habitats described according to type and size. Immature *Anopheles*, culicines and pupae from the described habitats were sampled using standard dipping methods to determine larval and pupae densities. Larvae were identified as anopheline or culicine, and counted. Pupae were not identified further. Binary logistic regression analysis was used to identify factors associated with the presence of immature *Anopheles* and culicines in each site.

**Results:**

A total of 1205 larval aquatic habitats were surveyed and yielded a total of 17,028 anopheline larvae, 26,958 culicine larvae and 1189 pupae. Peaks in larval abundance occurred in all sites in March–May and August-October coinciding with the rainy seasons. *Anopheles* larvae were found in 52.4% (n = 251) of aquatic habitats in Tororo, a site of high transmission, 41.9% (n = 536) of habitats in Kanungu, a site with moderate malaria transmission, and 15.8% (n = 418) in Jinja, a site with low malaria transmission. The odds of finding larvae was highest in rice fields compared to pools in both Tororo (odds ratio, OR = 4.21, 95% CI 1.22–14.56, p = 0.02) and Kanungu (OR = 2.14, 95% CI 1.12–4.07, p = 0.02), while in Jinja the odd were highest in containers (OR = 4.55, 95% CI = 1.09–19.14, p = 0.03). In Kanungu, larvae were less likely to be found in containers compared to pools (OR = 0.26, 95% CI 0.09–0.66, p = 0.008) and river fringe (OR = 0.19, 95% CI 0.07–0.52, p = 0.001). Medium sized habitats were associated with high odds of finding larvae compared to small habitats (OR = 3.59, 95% CI 1.18–14.19, p = 0.039).

**Conclusions:**

These findings show that immature *Anopheles* and culicines were common in areas of high and moderate transmission but were rare in areas of low transmission. Although immature *Anopheles* and culicines were found in all types of water bodies, they were most common in rice fields and less common in open drains and in river fringes. Methods are needed to reduce the aquatic stages of anopheline mosquitoes in human-made habitats, particularly rice fields.

## Background

Between 2000 and 2015 there has been remarkable progress in malaria control in sub-Saharan Africa mainly due to the massive deployment of insecticide-treated nets (ITNs), indoor residual spraying (IRS) and prompt treatment with artemisinin-based combinations [1]. It is clear though that in many places this combination of interventions is not sufficient, especially when addressing outdoor transmission. The World Health Organization (WHO) has called for new approaches using the most effective tools in a more targeted way to prevent disease and save lives in countries hardest hit [2].

In Uganda, pyrethroid resistance is widespread and likely to undermine the impact of ITNs that use unmodified pyrethroids [3–7]. *Anopheles* mosquitoes have also developed resistance to some of the insecticides commonly used IRS [7]. Malaria control may be further hindered if large-scale deployment of ITNs and IRS changes vector behavior from biting indoors to outdoors, biting times and species composition [8, 9]. As a result, there is a need for alternative control interventions to reduce the force of malaria infection.

In the last decade, WHO has promoted the Integrated Vector Management (IVM) approach through its Global Vector Control Response 2017–2030 [2, 10]. In this multi-sectoral approach, multiple control tools are combined to improve their efficacy, cost-effectiveness and sustainability. LSM targets immature mosquito populations by removing standing water, flushing aquatic habitats, or adding insecticides, microbial larvicides or natural predators to standing water to kill larvae [11–13]. Adult *Anopheles* control, complemented by larval control can significantly reduce malaria transmission in sub-Saharan Africa [13–16] and has been recommended to reduce outdoor transmission [17]. LSM has been incorporated in Integrated Vector Management (IVM) as a malaria control policy in Uganda [18] and scaling LSM in Uganda is highly recommended [19].

Effective implementation of LSM requires knowledge about *Anopheles* habitats. Malaria vector control programmes in Uganda have mainly targeted adult stages of the vector. Because of this less attention has been given to studying and characterizing habitats of immature *Anopheles* stages.

Although malaria control in Uganda has relied heavily on adult vector control, few studies have characterized *Anopheles* aquatic habitats in the country over the past 20 years. This study was designed to describe key *Anopheles* and culicine larval habitats and factors associated with larval abundance at three sites of varying malaria transmission in Uganda. The results of this study should be useful in planning and implementing larval management strategies.

## Methods

### Study sites

**Fig. 1:**
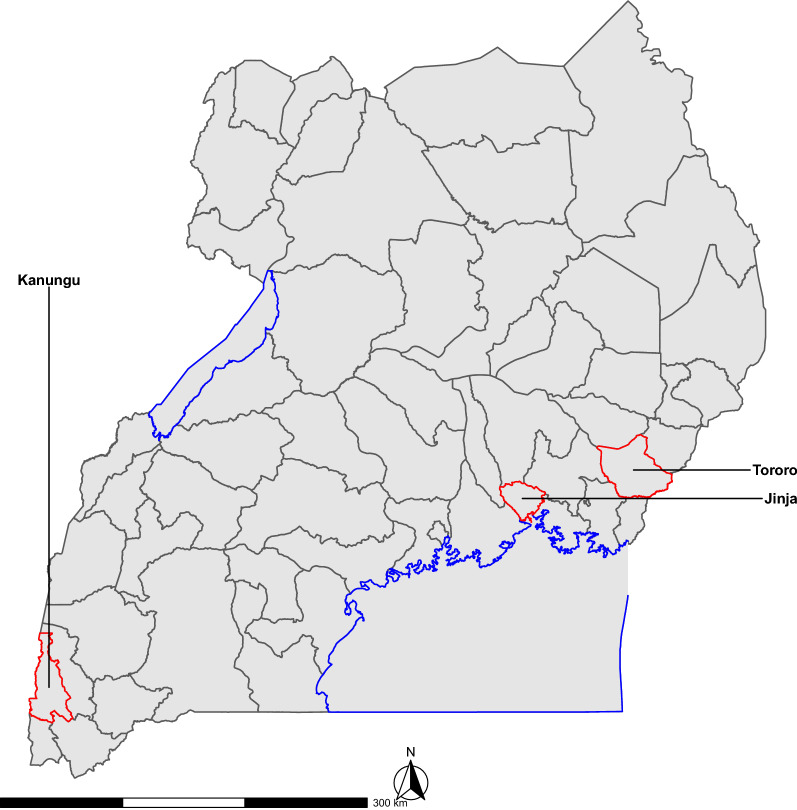
Map of Uganda showing the three study three sites; Jinja, Kanungu and Tororo

The study was carried out in two rural sub-counties (Nagongera and Kihihi) and one peri-urban sub-county (Walukuba) (Fig. 1). Nagongera sub-county is located in Tororo district (00° 46′ 10.6″, N 34° 01′ 34.1″ E). At the time the study was initiated, Tororo was an area of intense malaria transmission [20], although transmission has been greatly reduced following the implementation of IRS starting in December 2014 [8, 21, 22]. Tororo is situated at an elevation of 1185 m above sea level, and houses are constructed on low-lying hills. It is an area with savannah grassland interrupted by bare rocky outcrops and low-lying wetlands. Unproductive sandy soils are the most common, which tempts farmers to cultivate in and around wetlands specifically rice growing [23]. Other crops grown include; maize, cassava, sweet potatoes, sorghum, groundnuts, soya beans, beans, and millet. At the time the study was initiated, the major malaria vector species in Tororo were *Anopheles gambiae* sensu stricto (s.s.) and *Anopheles funestus* with small numbers of *Anopheles arabiensis* [24].

Kihiihi is one of the sub-counties in Kanungu district (00° 45′ 03.1″ S, 29° 42′ 03.6″ E). Kanungu is an area of moderate malaria transmission [20]. It is situated in rolling hills at an elevation of 1310 m above sea level. The main activity in Kanungu is agriculture, where farmers grow bananas, millet, rice, cassava, potatoes, sweet potatoes, tomatoes, maize, groundnuts, and beans. The main vector is *An. gambiae* s.s. [24]. Walukuba is a peri-urban sub-county in Jinja district (00° 26′ 33.2″ N, 33°13′ 32.3″ E). Jinja is a town with low malaria transmission and is situated at an elevation of 1215 m above sea level, close to a swampy area near Lake Victoria [20]. The major malaria vector species found here is *An. arabiensis* [3]. There are typically two rainy seasons in Uganda (March–May and August-October) with annual rainfall of 1000–1500 mm.

### Habitat definitions

Aquatic habitats were defined using a method that was described previously in the Gambia [25]: (1) freshwater marsh (swamp), was a large water body containing vegetation and tall papyrus, (2) river fringe, was the shallow edge of a permanent stream, associated with grass and tall reeds in deeper parts, (3) puddle was a small natural water-filled depression, (4) pool was a large man-made depression holding water, (5) water channel was an open flowing water used for irrigation, (6) foot print was a depression made by the foot of a person, cow or other animal where water collects, often associated with edges of large water bodies, (7) tire track was a water-filled depression made by a vehicle, (8) artificial pond was a large human-made permanent water body, (9) sand pit was a depression made after extraction of sand or bricklaying, (10) container was a discarded plastic or metal waste, (11) pit latrine was any hole used as a toilet containing water, (12) rice field was a flooded area used to grow rice, and (13) open drain was man-made and constructed for the purpose of getting rid of water (14) Lake fringe was the shallow edge of a lake, (15) flood water was a large natural water-filled depression a rising especially after heavy rains.

### Larval surveys

As part of ongoing cohort studies, 100 households were enrolled in each of the three study sites. The households were used as starting points for making transects. From each household, a transect 20 m wide was walked downhill until a maximum length of approximately 1 km or until a large permanent aquatic habitat was reached. Larval surveys were carried out using classical larval prospection in 3–5 transects per site per month to assess the presence of potential water bodies in transects. Potential larval habitats were described morphologically (type and size) as defined above and geo-referenced using a GPS device (Garmin GPS series GPSMAP^®^62.2.3). Purposeful sampling was done to maximize collection of the aquatic stages of mosquitoes. All aquatic habitats were sampled for the presence of anopheline and culicine larvae and pupae. Once viewed, mosquito larvae and pupae were collected using a 350 ml dipper (Clarke Mosquito Control Products, Roselle, IL). Plastic transfer pipettes were used to collect larvae and pupae in very small habitats where dippers could not be used. At each water body, a maximum of 10 dips were made to sample locations likely to harbour mosquito larvae, such as around tufts of submerged vegetation or substrate, edges of water bodies, and around floating debris. If transects included a water channel, river, or stream (long habitats) then measurements were made every 10 m along the water body. To avoid making several collections from the same habitat, a maximum of 2 measurements were made and, therefore, up to 20 dips per long habitat were sampled for mosquito larvae. The size of the water body was estimated visually and grouped into < 10 m in perimeter (small habitats), 10–100 m in perimeter (medium habitats), or > 100 m in perimeter (large habitats). Water from aquatic habitats collected by dippers was emptied into a white basin and checked for mosquito larvae and pupae. Specimens were identified morphologically to genus level, using the anopheline larvae morphological identification keys developed by Holstein in 1949 [26]. Mosquito larvae were recorded as anopheline or culicine and either early (L1–L2) or late (L3–L4) instars. The pupae were not identified further. The finding of at least one larva or pupa was sufficient to record a larval habitat as occupied (effective breeding site); no further quantitative estimates were made.

### Rainfall data

Monthly rainfall data was obtained for the period of February 2012 to January 2015 for Jinja, October 2011 to May 2013 for Kanungu and February 2012 to August 2013 for Tororo. The data were obtained from the NASA Tropical Rainfall Measuring Mission project [27]. The data were aggregated by site and averaged monthly for the three sites.

### Data management and analysis

Data were double entered into a Microsoft Access database and analysed using R statistical software [28]. Binary logistic regression analysis was used to determine variables associated with the presence or absence of *Anopheles* larvae at the three sites. Presence of *Anopheles* larvae was used as the dependent variable and habitat size and habitat type as the independent variables. Initial analyses indicated that late-instar *Anopheles* larvae counts were strongly correlated with early instar counts (r^2^ = 0.833, *p *< 0.001) at all three sites, and these data were pooled together for further analysis. Pools were used as baseline habitats since they appeared in considerable numbers in all the three sites. To determine the habitats most productive for larvae, habitats in which *Anopheles* and culicine larvae were found were expressed as percentages of all habitats sampled.

The relative abundance of *Anopheles* per habitat was calculated as the number of larvae divided by the number of dips taken from each larval habitat. Regression analysis was used to determine factors affecting larval relative abundance (y) after log-transforming log10 (y + 0.5) to stabilize the variance and improve normality of distribution. Correlation analysis was used to investigate the relationship between anopheline and culicine larvae and between *Anopheles* early and late instars in aquatic habitats. Statistical significance was set at a *p* value of < 0.05.

## Results

### Larval habitat types identified

**Fig. 2:**
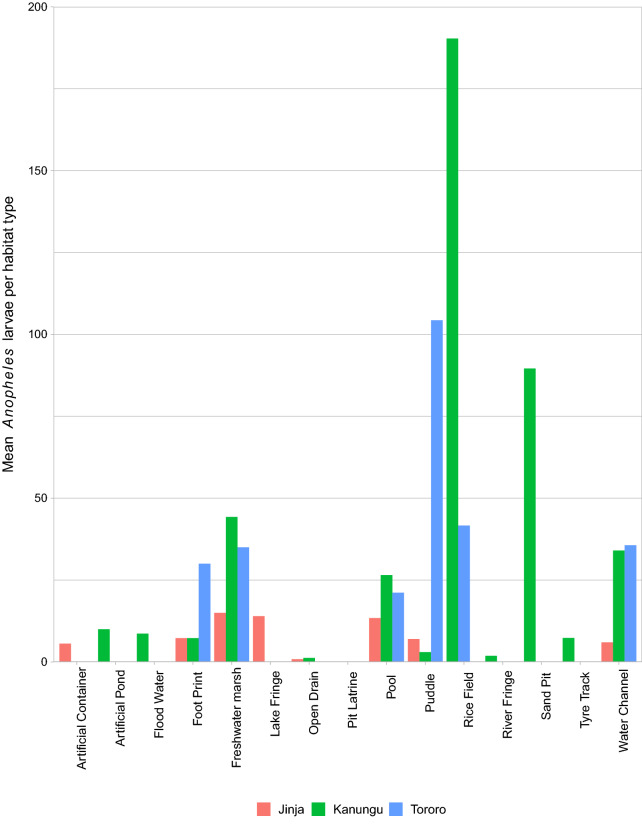
Showing the mean *Anopheles* larval abundance of habitats at three sites

The results of larval habitats surveyed and the number of larval habitats classified by one of fifteen types are presented in Fig. 2. The habitat types varied from site to site, but since pools were common in all three sites Tororo (n = 13), Kanungu (n = 62) and Jinja (n = 64), they were used as a reference habitat in the analysis. In Jinja, the most common aquatic habitats were water channels 42.1% (n = 176) and pools 15.3% (n = 64), in Kanungu water channels 23.3% (n = 125) and freshwater marshes 19.4% (n = 104) were the most common, while in Tororo, rice fields 40.6% (n = 112) and water channels 40.2% (n = 101) were the most common.

### Abundance of *Anopheles* larvae in aquatic habitats

The proportion of aquatic habitats found with *Anopheles* larvae varied significantly according to site (p < 0.001). A total of 1205 aquatic habitat types were characterized and sampled for mosquito larvae and pupae: 251 habitats in Tororo, 536 in Kanungu and 418 in Jinja (Table 1). A total of 17,028 anopheline larvae, 26,958 culicine larvae and 1189 pupae were collected at the three sites. Stratified by site; Jinja had 436 anopheline larvae, 1635 culicine larvae and 374 pupae. Kanungu had 11,257 anopheline larvae, 15,265 culicine larvae and 164 pupae, while Tororo had 5335 anopheline larvae, 4622 culicine larvae and 651 pupae.

**Table 1:** Distribution of different aquatic habitats at three sites in Uganda

Habitat type	Site			Total
	Jinja	Kanungu	Tororo	
Container	10	43	0	53
Artificial pond	1	18	2	21
Flood water	10	6	1	17
Foot print	27	24	4	55
Lake fringe	18	0	1	19
Open drain	45	24	5	74
Pit latrine	2	1	0	3
Pool	64	62	13	139
Puddle	43	9	7	59
Rice field	1	39	112	152
River fringe	4	59	0	63
Sand pit	0	14	1	15
Fresh water marsh	6	104	4	114
Tire track	11	8	0	19
Water channel	176	125	101	402
Total	418	536	251	1205

*Anopheles* larvae were found in 15.3% (64/418) of aquatic habitats in Jinja. The most productive habitats for *Anopheles* larvae were foot prints and containers in which *Anopheles* larvae were found in 22.2% (n = 27) and 20% (n = 10), respectively of aquatic habitats sampled. The most productive habitats for culicine larvae were open drains and containers in which culicine larvae were found in 28.9% (n = 45) and 40% (n = 10), respectively of aquatic habitats sampled (Table 2).

**Table 2:** Prevalence and density of larvae and pupae in different habitat types in Jinja district

Habitat type	N	% with *Anopheles*	% < 10 m perimeter	% with culicine	*Anopheles* density	Culicine density	Pupae density
Container	10	20.0	90.0	40.0	0.3	0.9	0.3
Artificial pond	1	0.0	100.0	0.0	0.0	0.0	0.0
Flood water	10	0.0	60.0	0.0	0.0	0.0	0.0
Foot print	27	22.2	100.0	14.8	0.2	0.1	0.0
Fresh water marsh	6	16.7	33.3	16.7	0.2	0.1	0.0
lake fringe	18	16.7	5.6	11.1	0.2	0.1	0.1
open drain	45	2.2	91.1	28.9	0.0	1.9	0.3
pool	64	12.5	92.2	14.1	0.3	0.5	0.1
puddle	43	2.3	93.0	2.3	0.0	0.0	0.0
Rice field	1	0.0	0.0	0.0	0.0	0.0	0.0
river fringe	4	0.0	50.0	0.0	0.0	0.0	0.0
Tyre track	11	0.0	100.0	0.0	0.0	0.0	0.0
Water channel	176	6.8	48.3	5.7	0.1	0.1	0.0

*Anopheles* larvae were found in 41.8% (224/536) of aquatic habitats in Kanungu. The most productive habitats for *Anopheles* larvae were rice fields and freshwater mashes in which *Anopheles* larvae were found in 71.8% (n = 39) and 67.3% (n = 104) respectively of aquatic habitats sampled. Likewise, the most productive habitats for culicine larvae were rice fields and freshwater mashes in which culicine larvae were found in 61.5% (n = 39) and 65.3% (n = 104) respectively of aquatic habitats sampled (Table 3).

**Table 3:** Prevalence and density of larvae and pupae in different habitat types in Kanungu district

Habitat type	N	% with *Anopheles*	% < 10 m perimeter	% with culicine	*Anopheles* density	Culicine density	Pupae density
Artificial pond	18	55.56	55.56	50.00	0.56	4.57	0.06
Container	43	13.95	95.35	13.95	0.00	0.09	0.00
Flood water	6	50.00	50.00	50.00	0.43	1.13	0.03
Foot print	24	45.83	95.83	33.33	0.33	1.16	0.00
Fresh water marsh	104	67.31	6.73	65.38	2.98	7.16	0.06
Open drain	24	33.33	54.17	29.17	0.04	6.62	0.01
Pool	62	41.94	69.35	32.26	1.11	0.97	0.00
Puddle	9	22.22	100.00	22.22	0.07	0.16	0.00
Rice field	39	71.79	17.95	61.54	13.66	4.54	0.14
River fringe	59	15.25	47.46	11.86	0.03	0.08	0.00
Sand pit	14	35.71	57.14	35.71	3.20	1.52	0.00
Tyre track	8	37.50	100.00	37.50	0.28	1.98	0.00
Water channel	125	33.60	55.20	19.20	1.14	1.76	0.02

*Anopheles* larvae were found in 52.6% (132/251) of aquatic habitats in Tororo. The most productive habitats for *Anopheles* larvae were rice fields and pools in which *Anopheles* larvae were found in 70.5% (n = 112) and 46.4% (n = 13), respectively of aquatic habitats sampled. Likewise, the most productive habitats for culicine larvae were rice fields and pools in which culicine larvae were found in 57.1% (n = 112) and 46.1% (n = 13), respectively of aquatic habitats sampled (Table 4).

**Table 4:** Prevalence and density of larvae and pupae in different habitat types in Tororo district

Habitat type	N	% with *Anopheles*	% < 10 m perimeter	%with culicine	*Anopheles* density	Culicine density	Pupae density
Artificial pond	2	0.00	50.00	0.00	0.00	0.00	0.00
Flood water	1	0.00	100.00	0.00	0.00	0.00	0.00
Foot print	4	25.00	100.00	25.00	0.75	2.55	0.00
Fresh water marsh	4	25.00	25.00	25.00	0.88	0.10	0.00
Open drain	5	0.00	80.00	0.00	0.00	0.00	0.00
Pool	13	46.15	84.62	46.15	0.98	1.77	0.03
Puddle	7	42.86	100.00	42.86	4.47	3.01	0.47
Rice field	112	70.54	82.14	57.14	2.98	3.24	0.54
Sand pit	1	0.00	100.00	0.00	0.00	0.00	0.00
water channel	101	41.58	97.03	24.75	1.48	0.44	0.01

Human-made habitats were the most contributors of *Anopheles* larvae at all the three sites. In Jinja, containers (n = 10), foot prints (n = 27) and pools (n = 64) were among the top five of aquatic habitats found with *Anopheles* larvae with proportions of 20%, 22.2% and 12.5% respectively. In Kanungu, rice field (n = 39), artificial ponds (n = 18) and foot prints (n = 24) were among the top five of aquatic habitats found with *Anopheles* larvae with proportions of 71.8%, 55.2% and 45.8% respectively. In Tororo, rice field (n = 112), pools (n = 46.4) and foot prints (n = 4) were among the top five of aquatic habitats found with *Anopheles* larvae with proportions of 70.5%, 46.4% and 25%, respectively (Tables 2, 3, 4).

Larval densities for each habitat type and size at the three sites were highly variable (Tables 5, 6, 7). In Jinja, high larval densities were found in open drains and medium sized habitats with larval densities of 1.7 (0.81–2.73) and 0.81(0.23–1.39) respectively. In Kanungu high larval densities were found in rice fields and large sized habitats with larval densities of 8.63 (6.99–9.26) and 2.09 (1.47–2.71), respectively, while in Tororo high larval densities were found in puddles and medium sized habitats with larval densities of 3.07 (1.61–4.54) and 2.24 (1.43–3.04), respectively.

**Table 5:** Mean adult larval densities per habitat characteristic and odds ratio for presence vs. absence of larvae in habitat in comparison to pools for Jinja district

	Larval density (per dip)			Regression analysis			
	Mean	95% CI		OR	95% CI		p-value
Habitat type							
Pool	1.023	0.082	1.963	1			
Container	0.590	− 0.490	1.670	4.548	1.099	19.139	0.034
Foot print	0.271	− 1.323	0.781	1.518	0.500	4.378	0.445
Fresh water marsh	0.593	− 1.261	2.448	0.840	0.040	6.367	0.881
Lake fringe	0.790	− 0.436	2.016	1.485	0.241	7.763	0.648
Open drain	1.768	0.812	2.725	1.686	0.680	4.213	0.258
Puddle	0.189	− 1.275	1.653	0.227	0.034	0.898	0.062
Water channel	0.087	− 0.899	0.724	0.500	0.211	1.199	0.115
Habitat size							
< 10 m perimeter	0.501	0.059	0.943	1			
> 100 m perimeter	0.408	− 1.584	2.399	0.363	0.045	1.782	0.266
10–100 m perimeter	0.814	0.233	1.395	1.096	0.493	2.383	0.819

*CI* 95% confidence interval, *OR* odds ratio

**Table 6:** Mean adult larval densities per habitat characteristic and odds ratio for presence vs. absence of larvae in habitat in comparison to pools for Kanungu district

	Larval density (per dip)			Regression analysis			
	Mean	95% CI		OR	95% CI		p-value
Habitat type							
Pool	0.981	0.325	1.638	1			
Artificial pond	1.274	0.230	2.319	1.344	0.435	4.156	0.603
Container	0.334	− 0.994	1.661	0.255	0.086	0.664	0.008
Foot print	1.298	0.282	2.314	1.328	0.503	3.482	0.562
Fresh water marsh	0.800	0.354	1.246	2.043	0.966	4.377	0.063
Open drain	0.519	− 0.598	1.637	0.669	0.237	1.778	0.429
Rice field	8.626	6.991	9.262	2.143	1.213	4.075	0.023
River fringe	0.014	− 1.162	0.934	0.192	0.078	0.523	0.001
Sand pit	1.587	0.183	2.990	0.750	0.208	2.458	0.642
Water channel	1.466	0.939	1.992	0.659	0.349	1.246	0.197
Habitat size							
< 10 m perimeter	0.068	− 0.481	0.346	1			
> 100 m perimeter	2.089	1.469	2.708	1.747	0.929	3.274	0.082
10–100 m perimeter	0.910	0.460	1.361	1.485	0.912	2.419	0.111

*CI* 95% confidence interval, *OR* odds ratio

**Table 7:** Mean adult larval densities per habitat characteristic and odds ratio for presence vs. absence of larvae in habitat in comparison to pools for Tororo district

	Larval density (per dip)			Regression analysis			
	Mean	95% CI		OR	95% CI		p-value
Habitat type							
Pool	1.912	0.842	2.981	1			
Foot print	2.905	0.464	5.347	0.470	0.020	4.974	0.558
Fresh water marsh	1.118	− 1.342	3.578	0.187	0.005	2.723	0.259
Puddle	3.074	1.610	4.538	1.058	0.151	7.007	0.953
Rice field	1.559	1.096	2.021	4.212	1.225	14.557	0.028
Water channel	1.396	0.786	2.006	0.967	0.293	3.333	0.956
Habitat size							
< 10 m perimeter	1.669	1.015	2.323	1			
> 100 m perimeter	2.076	0.701	3.451	2.947	0.357	71.828	0.387
10–100 m perimeter	2.237	1.428	3.047	3.588	1.177	14.185	0.039

*CI* 95% confidence interval, *OR* odds ratio

### Habitat sizes and its contributions to larval abundance

**Fig. 3:**
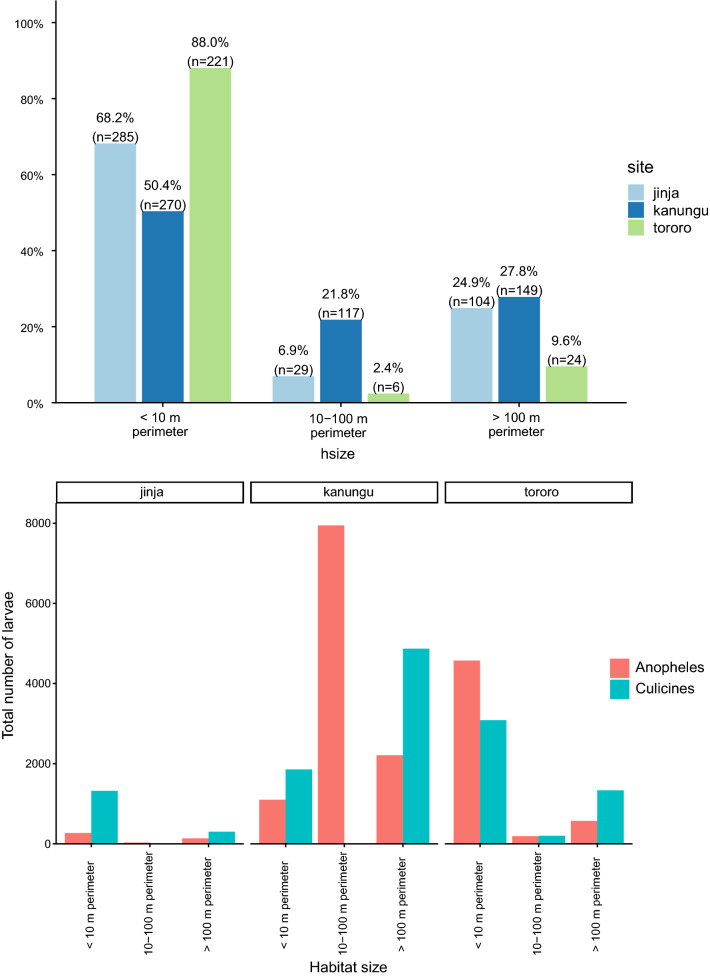
Showing contribution of different habitat sizes to *Anopheles* and culicine larvae abundance

Small habitats of < 10 m in perimeter were the most common aquatic habitats at all sites (Fig. 3).

In Jinja, 68.2% (285/418) of the aquatic habitats found were small (< 10 m in perimeter). More than 90% of three out of five of most productive habitats for *Anopheles* larvae found were small consisting of containers 90% (n = 10), foot prints 100% (n = 27) and pools 92.2% (n = 64). In Kanungu, 50.4% (270/536) of the aquatic habitats found were small (< 10 m in perimeter). More than 55% of three out of five of most productive habitats for *Anopheles* larvae found were small consisting of foot print 95.8% (n = 24), artificial ponds 55.6% (n = 18) and pools 69.5% (n = 62). In Tororo, 88.0% (221/251) of the aquatic habitats found were small (< 10 m in perimeter). More than 80% of four out of five of most productive habitats for *Anopheles* larvae found were small consisting of rice fields 81.3% (n = 112), foot prints 100% (n = 4), pools 84.7% (n = 13), water channels 97.0%, (n = 101) and foot prints 25% (n = 4).

Despite these habitats being most prevalent, the mean *Anopheles* larvae count in different habitat sizes varied from site to site. In Tororo and Jinja, higher larvae count per habitat were obtained in small habitats (< 10 m in perimeter) followed by medium habitats (10–100 m in perimeter), while in Kanungu, higher larvae count per habitat were obtained in large habitats (> 100 m in perimeter) followed by medium habitats (Fig. 3).

### Effect of rainfall on immature *Anopheles* and culicines abundance

**Fig. 4:**
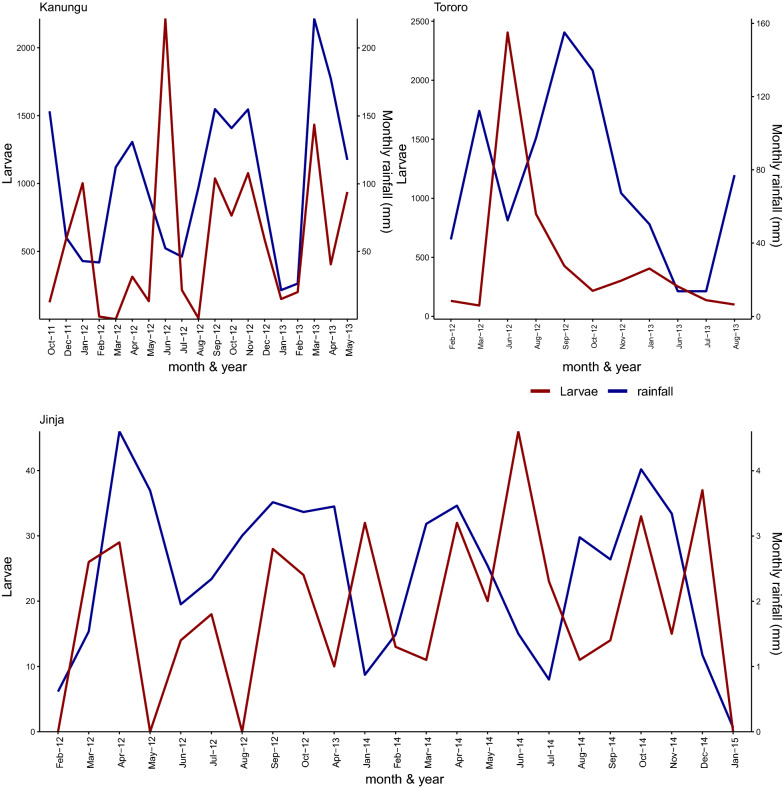
Showing relationship between rainfall and abundance of immature *Anopheles* and culicines

The relationship between larval abundance and rainfall, are presented in Fig. 4. Overall, there was no clear relationship between rainfall and number of larvae found in the habitats at all sites. Important to note is that dry months (January-March and June–August) yielded low numbers of *Anopheles* larvae.

### Correlation between early (L1–L2) and late (L3–L4) stages of anopheline and culicine larvae in habitats

**Fig. 5:**
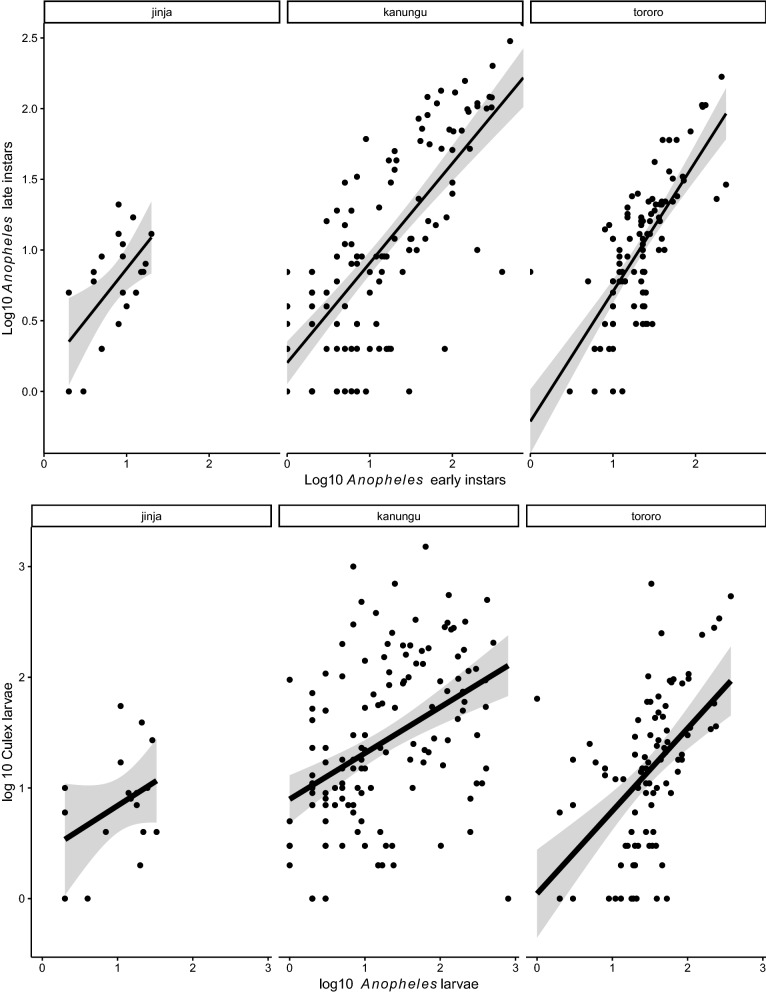
Showing association between early and late *Anopheles* instars and *Anopheles* and culicine larvae in aquatic habitats

Linear association between early and late instars of anopheline and culicine larvae in aquatic habitats and are shown in Fig. 5. There was considerable differences in sites; in Jinja, there was a weak positive log linear association between the early and late instars *Anopheles* larvae in the same aquatic habitat (r^2^ = 0.31, df = 62, p < 0.001), in Kanungu and Tororo, the association was strong (Kanungu: r^2^ = 0.69, df = 222, p < 0.001: and Tororo: r^2^ = 0.59, df = 131, p < 0.001).

In contrast, there was a positive but weak log linear association between the anopheline and culicine larvae in the same aquatic habitats in Jinja (r^2^ = 0.32, df = 62, p = 0.005), but not Kanungu (r^2^ = 0.47,df = 222, p = 0.23) and Tororo (r^2^ = 0.55, df = 131, p < 0.001).

## Discussion

Understanding habitat ecology such as the most productive habitat, habitat size and habitat type helps with larval control programmes. Larval control programmes may include larviciding or alternative methods of control, e.g. improving drainage ditches, filling sand pits, and other means of making habitats unavailable, which vary by habitat type or size. This study compared ecologies of mosquito larvae at sites of varying malaria transmission intensities in Uganda. Habitat types were driven by the economic activity of the sites and thus human made and small habitats of < 10 m in perimeter contributed to most of aquatic habitats found with immature *Anopheles* and culicines. Furthermore, anophelines and culicines always occupied the same aquatic habitats and were influenced by rainfall.

A broad diversity of aquatic habitats was surveyed in the study sites. There was a considerable differences in aquatic habitats with anopheline larvae found ranging from 22.2% (n = 27) for footprints in Jinja, 71.8% for rice fields in Kanungu (n = 39) and 70.5% (n = 112) for rice field in Tororo. However, these figures should be interpreted with caution since sampling dates and seasons were variable hence direct comparisons could not be made. Although *Anopheles* larvae were found in 22.2% of footprints sampled in Jinja, an area of low transmission, water channels accounted for most of the *Anopheles* larvae collected because they were the most common habitats (n = 176). Jinja is a semi-urban area located near the shores of Lake Victoria and, therefore, likely to have many water channels due to water flowing from shores of Lake Victoria. These channels therefore have constant supply of water throughout the year that favors larvae growth. Likewise, most *Anopheles* larvae collected in Kanungu were from freshwater marshes because they were the most abundant (n = 104). These fresh water marshes rarely dry out and therefore act as permanent breeding sites for *Anopheles* throughout the year. In Tororo district, an area of intense transmission, most larvae were collected in rice fields (n = 112). The differences in habitat types by site could be partly explained by differences in economic activities and geography of these sites. Kanungu is a low-lying area with many swamps and rivers as well as rice growing; and in Tororo rice growing is common and there is always need to divert water from rivers to the rice fields to support rice growing especially during the dry season.

The prevalence of immature *Anopheles* and culicines in aquatic habitats in the three sites mirrored malaria transmission reported at these sites [20, 29]. Jinja an urban area with low malaria transmission, had the lowest proportion of aquatic habitats found with *Anopheles* larvae (15.3%), Kanungu a rural area, with moderate transmission, immature *Anopheles* and culicines were found in close to half of aquatic habitats surveyed (41.9%), while Tororo district, is a rural area of high malaria transmission, immature *Anopheles* and culicines were found in slightly more than half of the aquatic habitats surveyed (52.4%). This could probably be due to number of larvae in aquatic habitats translate into adult mosquito densities and therefore the relation between larval densities, adult mosquito densities and malaria transmission. Epidemiological, entomological and parasitological studies have demonstrated similar trends using test positivity rates (TPR) of malaria parasites and daily human biting rates by adult *Anopheles* mosquitoes collected in these study area [20, 29–31].

A particularly important finding is the key role of rice fields in the production of immature *Anopheles* and culicines as observed in both rural areas. Rice fields produce prodigious numbers of immature *Anopheles* and culicines [32, 33], but this does not necessarily lead to increased malaria transmission [32]. Rice growing in Kihiihi and Nagongera is a well-known agricultural activity in these areas and is supported in the national plan [23, 34] and farmers in these rice growing areas often divert water from streams and rivers into their gardens with the aim of supporting rice growing in the dry season. This in turn creates puddles and small, clear open habitats within the rice field that are favorites for *An. gambiae* sensu lato (s.l.) [35]. Farmers grow rice two seasons a year in these areas and this creates aquatic habitats all year round, extending the transmission season.

The aquatic habitats in these areas were much varied in number and composition which made direct comparisons of larval abundance between sites difficult. In addition, the number of habitats sampled varied between habitat types hence results should be interpreted cautiously. Even though rice fields presented much a risk factor for immature *Anopheles* and culicines breeding in both Kanungu and Tororo, it is important to emphasize that rice fields were by far the most common aquatic habitats. In Jinja and Tororo, the habitats associated with higher odds of finding larvae did not necessary have higher larvae densities.

Human-made habitats such as borrow pits, puddles and rice fields accounted for more than 50 *Anopheles* larvae per habitat per sampling and were thus the most productive habitats (Fig. 2). In Kanungu, brick laying and sand mining is a common activity and therefore this creates borrow pits that contain water throughout the year. This would also lead to an extension of malaria transmission throughout the year. Human activities like fishing, agriculture and brick laying have been previously reported to play important roles in creating habitats for mosquito larvae [36–38].

In the future, as the population of Uganda grows, there is likely to be an increase in agriculture and house construction, favouring the creation of new aquatic habitats for *Anopheles*. It is important to adapt larval source management (LSM) strategies to reduce the number of vectors produced from these habitats. Rainfall occurs to a greater or lesser extent throughout the year and immature *Anopheles* and culicines were common in the three study sites throughout much of the year. There was no strong relationship between rainfall and immature *Anopheles* and culicines numbers indicating that during periods of low rainfall mosquitoes continue to thrive in semi-permanent or permanent water bodies. Although targeting LSM when aquatic are few has been recommended [14], this analysis suggests that all water bodies need to be treated with larvicides and environmental management directed at particular habitats.

Small aquatic habitats (less than 10 metres in diameter) were the main source of immature *Anopheles* and culicines collected at all sites, followed by medium aquatic habitats (10–100 m in perimeter) and lastly by large aquatic habitats (> 100 m in perimeter). This is partly good news as most small aquatic habitats are unstable and likely to dry out compared to large aquatic habitats. Unstable aquatic habitats have been shown to be less efficient in maintaining malaria transmission in western Kenya and Tanzania [39–41]. However, in these sites aquatic habitats mainly man-made are likely to be refilled by diverting waters from rivers and stream for purposes of supporting agriculture. This would maintain malaria transmission throughout the year.

Immature *Anopheles* and culicines often occurred in the same water bodies. This suggests that the same aquatic habitats targeted for *Anopheles* larval control programmes could also be targeted for culicine larvae control programmes. Previous studies have shown that *Anopheles* and culicine larvae are likely to occur in the same habitats [42]. Likewise*, Anopheles* early and late instars were highly correlated at the three sites.

There were limitations to this study. Firstly, *Anopheles* mosquitoes were not identified to species level. Analysis of adult mosquitoes collected in these areas in the same periods that larval surveys were done indicates that most adults were *An. gambiae* s.l. Out of all *Anopheles* mosquitoes caught, 88.5% of *Anopheles* mosquitoes were *An. gambiae* s.l. in Jinja, 99.8% in Kanungu and 93.5% in Tororo [8, 20]. Secondly, counts from larval surveys do not estimate the number of larvae produced from the different habitats according to surface area of each habitat. So, although one small puddle may have high numbers of larvae, a rice field nearby with lower densities but larger surface area could be producing several orders of magnitude more larvae than the puddle simply because it is so much bigger.

## Conclusions

Immature *Anopheles* and culicines occurred throughout the year in a wide range of water bodies, many of them human-made. Rice fields were particularly important sources of immature *Anopheles* and culicines. Larval control programmes would need to treat all aquatic habitats and use environmental management to reduce the force of malaria infection.

## Data Availability

Data are available upon reasonable request by an email to the corresponding author.

## Abbreviations

ACT: Artemisinin-based combination therapy
aEIR: Annual entomological inoculation rates
GPS: Global positioning systems
IRS: Indoor residual spraying
IVM: Integrated Vector Management
LLIN: Long-lasting insecticidal nets
LSM: Larval Source Management
WHO: World Health Organization
